# Intraventricular Hemorrhage after Epidural Blood Patching: An Unusual Complication

**DOI:** 10.1155/2014/406289

**Published:** 2014-12-04

**Authors:** Mohammad Sorour, Khaled M. Krisht, William T. Couldwell

**Affiliations:** Department of Neurosurgery, Clinical Neurosciences Center, University of Utah, 175 N. Medical Drive East, Salt Lake City, UT 84132, USA

## Abstract

The authors present two cases of intraventricular hemorrhage (IVH) believed to be a result of epidural blood patching. The first was a 71-year-old woman who had new onset of nontraumatic IVH on computed tomography (CT) scan after undergoing an epidural blood patch (EBP). This amount of intraventricular blood was deemed an incidental finding since it was of very small volume to account for her overall symptoms. The second patient, a 29-year-old woman, was found to have nontraumatic IVH three days after undergoing an EBP. This was seen on CT scan of the head for workup of pressure-like headaches, nausea, vomiting, and absence seizures. Conservative management was followed in both instances. Serial CT scan of the head in our first patient displayed complete resolution of her IVH. The second patient did not have follow-up CT scans because her overall clinical picture had improved significantly. This highlights a potential sequel of EBP that may be observed on CT scan of the head. In the event that IVH is detected, signs and symptoms of hydrocephalus should be closely monitored with the consideration for a future workup if warranted by the clinical picture.

## 1. Introduction

Epidural blood patch (EBP) is considered the most effective treatment for refractory postdural puncture headache (PDPH) [1–3], which can result from cerebrospinal fluid (CSF) leakage at the dural puncture site [2, 4]. Autologous blood is injected into the patient's epidural space, tamponading the dorsal dural defect, preventing further CSF leakage [2, 5]. The procedure carries low rates of complications [1, 6], which are usually mild and self-limiting [3]. Serious complications have been described in the literature in exceedingly low rates. We describe two cases of iatrogenic intraventricular hemorrhage (IVH) as an unusual complication of EBP.

**Case 1 .**

**Figure 1:**
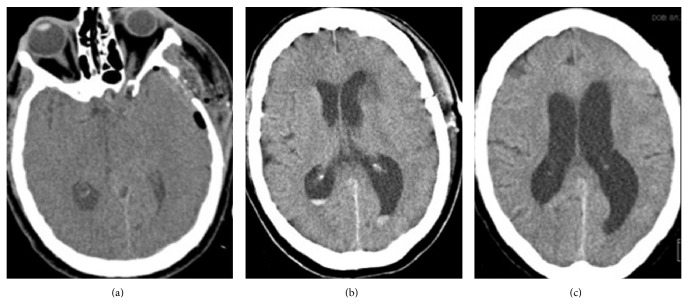
(a) Noncontrast axial computed tomography scan of the head done on postoperative day 5 showing some postoperative changes after a left frontotemporal craniotomy for resection of the patient's recurrent planum sphenoidale meningioma. The scan did not show any intraventricular hemorrhage; however, it showed unilateral enlargement of the left lateral ventricle. (b) Noncontrast axial computed tomography scan of the head done one day after the epidural blood patch showing a new-onset hemorrhage layering the occipital horns of both lateral ventricles. (c) Noncontrast axial head computed tomography scan done two weeks later showing complete resolution of the intraventricular hemorrhage.

A 71-year-old woman underwent transsphenoidal resection of a planum sphenoidale meningioma for visual symptoms. Two years later, visual acuity in her left eye decreased. MRI demonstrated tumor recurrence with severe optic nerve compression. She underwent a left frontotemporal craniotomy for resection and placement of a lumbar drain, which was weaned on postoperative (POD) day 3. The patient then developed positional headaches consistent with a CSF leak. A CT scan showed expected postoperative changes, along with unexplained asymmetrical unilateral enlargement of the left lateral ventricle (Figure 1(a)).

After a trial of conservative management did not resolve the headaches, she underwent an EBP. A 17-gauge Tuohy needle was inserted midline at the L4-L5 level under fluoroscopic guidance until the epidural space was reached, and 30 mL of autologous blood was injected into the epidural space. The patient immediately experienced improvement in her headaches. The next day, a CT scan prompted by memory difficulty disclosed new intraventricular blood associated with unilateral hydrocephalus not evident on her routine postoperative scan (Figure 1(b)). Her coagulation profile was normal (INR = 1.1, PTT = 32 s). She was managed conservatively until her clinical symptoms and IVH were resolved on repeat CT scan a few days later (Figure 1(c)).

**Case 2 .**

A 29-year-old woman with a history of spastic cerebral palsy underwent placement of an intrathecal baclofen pump. The catheter was placed under intraoperative fluoroscopic guidance at the L3-L4 level, and clear CSF was noted to be draining. On POD 1, she developed positional spinal headaches and nausea suggesting a CSF leak. She was initially managed conservatively, but because symptoms worsened, she underwent EBP a week later. A 17-gauge Tuohy needle was used to access the epidural space at the L4-L5 level under fluoroscopic guidance. Eighteen milliliters of autologous blood was injected into the epidural space, and she experienced immediate improvement in her symptoms.

**Figure 2:**
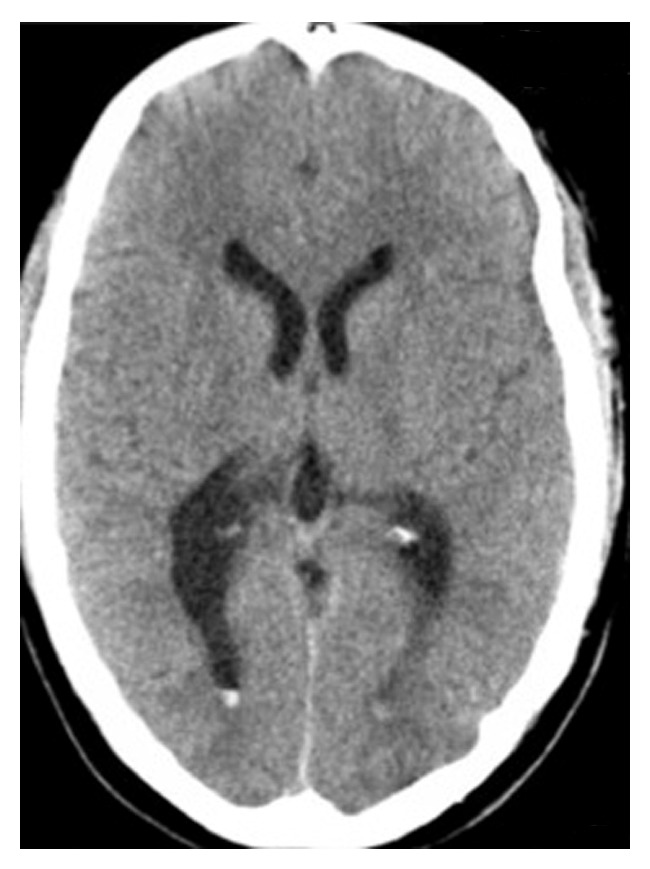
Noncontrast axial computed tomography scan of the head done 3 days after epidural blood patch showing a small amount of blood layering the occipital horns of both lateral ventricles.

Three days later, she developed new-onset, pressure-like, nonpositional headaches associated with episodes of staring into space, vomiting, and blurry vision lasting for 5–10 minutes and associated with right gaze deviation followed by confusion and restlessness. A noncontrast CT scan showed a small amount of IVH layering in her occipital horns along with small subarachnoid hemorrhage (SAH) adjacent to the right vertex (Figure 2). Her coagulation profile was normal (INR = 1.0, PTT = 27 s). She was started on levetiracetam (500 mg twice daily) for her seizures. She underwent long-term surface electroencephalogram monitoring with resolution of her seizure activity and clinical improvement. Because the volume of blood was minimal and her clinical status had improved, the patient did not undergo follow-up CT scans to document resolution of her IVH.

## 2. Discussion

EBP is a safe and effective method for treating PDPH, with an overall low rate of complications [2, 3, 5]. To our knowledge, there has been no previous description of EBP causing IVH. Hasiloglu et al. [5] reported a case of iatrogenic cranial SAH resulting from EBP in a patient with sudden onset of headache and neck pain from a spontaneous cervicothoracic spinal CSF leak. The SAH resulted from the passage of a large volume of injected blood (50 mL) from the epidural space into the subarachnoid space through the dura-arachnoid defect, the size of the dura-arachnoid defect, the lack of compliance of the epidural space, and the rate of injection. While that case involved the settling of blood within the cranial subarachnoid space, our patients demonstrated blood within the ventricular space. We believe that the source of the IVH was the autogenously injected blood rather than direct trauma from the epidural access and that the communication with the subarachnoid space was through the initial dura-arachnoid tear rather than from the EBP procedure. Although previous reports of inadvertent dural puncture from EBP have been reported [7], the use of real-time visualization under live fluoroscopy makes inadvertent dural tears unlikely. Our patients experienced immediate improvement of their postdural puncture symptoms, which indicates that the injected blood successfully tamponaded the dural defects in both cases. As in the case of Hasiloglu et al. [5], blood reached the subarachnoid space and continued into the ventricular system, likely through the foramina of Magendie or Luschka or both, causing blood to appear in the occipital horns.

Although IVH after EBP has not been reported in the neurosurgical literature, it may not be entirely rare. A small amount of IVH or SAH may not produce significant clinical symptoms and since many patients treated with EBP experience immediate improvement in their intracranial hypotension symptoms, cases may go undetected and therefore are likely underreported. In our patients, the small amount of occipital IVH on the CT scans was likely just an incidental finding. Our second patient's symptoms of seizures, headaches, and nausea are more in line with her cerebral palsy and less likely to be related to the small amount of occipital IVH.

The extent of the dura-arachnoid defect causing the CSF leak and the compliance of the epidural space may contribute to the development of IVH. It is reasonable to assume that the dura-arachnoid defect from insertion of a Tuohy needle is larger than that created by a lumbar needle, which may have contributed to the PDPH and blood leakage after the EBP procedure. Assuming the epidural space was noncompliant, this would partially explain the passive passage of blood from the epidural to the subarachnoid space. While we cannot verify the source of blood and explain its mode of transmission into the intraventricular space, the temporality of the EBP and CT head acquisition within a nontraumatic context, as well as the CT characteristics of the blood suggesting an early age (hyperdensity on head CT), provides a likely cause-effect explanation.

A volume of blood to be injected for an EBP between 10 and 20 mL appears to be suitable [2, 6], with overall success rates of 60–75% and low complication rates [2]. In both of our cases, the volume of blood used was not large, and we do not believe that the amount of the injected blood was a major determinant of the complication.

These cases demonstrate the potential for passage of blood through the subarachnoid space into the ventricular spaces after EBP. Although no serious complications resulted from the IVH, the neurosurgeon should be cognizant of this possibility and the potential for serious complications. Patients should not routinely undergo CT imaging after EBP; however, if IVH is incidentally detected on CT head as part of a workup for potential causes of new neurological symptoms or changes in level of alertness, the treating physician as well as the patient and family should be on alert for signs and symptoms of hydrocephalus since it can be a complicating factor. The mechanism of EBP spread is likely multifactorial, depending on the amount of injected blood, the size of the dura-arachnoid defect, and the compliance of the epidural space. A more objective way of ascertaining the exact mechanism of spread and establishing the effects of these factors could involve animal experiments with a red blood cell tracer to localize the spread of blood.
